# Seroprevalence of SARS-CoV-2 among Children Visiting a Tertiary Hospital during the Prevaccination Period, Southwest Region, Saudi Arabia

**DOI:** 10.3390/vaccines10081344

**Published:** 2022-08-18

**Authors:** Ali Alsuheel Asseri, Abdullah Alsabaani

**Affiliations:** 1 Department of Child Health, King Khalid University, Abha 62529, Saudi Arabia; 2 Department of Family and Community Medicine, College of Medicine, King Khalid University, Abha 62529, Saudi Arabia

**Keywords:** children, COVID-19, SARS-CoV-2, seroprevalence, COVID-19 serological testing

## Abstract

Background: In the early days of the COVID-19 pandemic, tests to ascertain whether individuals were infected with SARS-CoV-2 were often unavailable. One method to deal with this issue is to test for SARS-CoV-2 antibodies. This study sought to determine the seroprevalence of SARS-CoV-2 in children in Saudi Arabia before vaccines were available to them. Methods: This study was conducted among children who visited the tertiary Maternity and Children Hospital in Abha city, Saudi Arabia. Serum samples were screened for SARS-CoV-2-specific IgG, IgM, and IgA antibodies using ELISA. The crude and adjusted seroprevalence values among the studied children were calculated. Results: Among the 413 children studied, the ages of enrolled patients ranged from newborn to 12 years, with a median age of three years. We identified 127 (30.7%) seropositive children. IgG was exclusively positive in 43 (10.4%); IgM was exclusively positive in 8 (1.9%), and IgA was exclusively positive in 15 (3.6%) children. Conclusions: This study is the first to estimate the seroprevalence of SARS-CoV-2 among the pediatric population seeking medical care in southwestern Saudi Arabia. The findings shed light on the dynamics of virus transmission in the community and provide a good reference for future studies. Future research should examine factors related to SARS-CoV-2 infection and seroprevalence among pediatric populations.

## 1. Introduction

COVID-19 is a highly contagious respiratory disease caused by SARS-CoV-2. The COVID-19 pandemic, which began in 2020, has had a devastating impact globally. As of July 2022, the World Health Organization (WHO) estimated that there were over 500 million cases of the virus and over 6 million deaths worldwide [1]. Additionally, during the pandemic through 2021, the WHO estimated that there were nearly 15 million additional deaths caused by both the virus itself and the impact of the pandemic on other health outcomes [2]. As of July 2022, the WHO also estimated that there had been nearly 800,000 cumulative cases of COVID-19 and over 9000 COVID-19 deaths in the Kingdom of Saudi Arabia [1]. Both the global and Saudi-specific estimates for deaths and cases are likely underestimated as cases of the disease go unreported. [3].

Cases of COVID-19 among children have received attention because they are often a very vulnerable population. Although children have a much lower case-fatality rate for COVID-19 compared to older populations [4], preventing infections among this population remains an important goal. In Saudi Arabia, one study estimates that children account for about 8% of cases [5], but the actual number may be higher due to underreporting. Additionally, these numbers may be underestimated because children are more likely to have asymptomatic infections and therefore not get tested [6].

Antibody testing for COVID-19 is a tool that can be used to determine the proportion of a population that has been infected with SARS-CoV-2. Antibody tests identify antibodies for SARS-CoV-2, which, in the prevaccine era, would most likely be caused by infection with the virus [7]. These antibodies are also present in asymptomatic individuals. There are limitations to the usage of antibody tests for COVID-19; they are not useful for identifying an active infection, and they can also result in false-positive and false-negative results. Additionally, some antibodies are cross-reactive with other viruses, which means a positive test result may not be due to a COVID-19 infection, but rather due to an infection with other viruses [7,8].

Previously, antibody studies were used to assess the proportion of asymptomatic COVID-19 infections. A systematic review of these studies estimated that around one-third of SARS-CoV-2 infections are asymptomatic [9]. A previous nationwide SARS-CoV-2 study in Saudi Arabia found that about 11% of the population had antibodies. In this study, the seroprevalence was much higher than the reported COVID-19 infection rate, and the seroprevalence was higher among healthcare workers [10]. In February of 2022, the United States Centers for Disease Control published the seroprevalence of SARS-CoV-2 among children between 0 and 11 years of age ranging between 40 and 75% [11].

There have been some studies that examined the presence of antibodies among children. These previous studies reported different seroprevalence values, ranging from a low value of 1.3% in Northern Cyprus [12] to 5.8% among school- and preschool-age children in Canada [13], 9.5% and 15.4% (for IgG) in Italy [14,15], and a high of 25.3% in Brazil [16]. Further seroprevalence studies among children in diverse geographic locations can provide additional details on SARS-CoV-2 infection among children. Therefore, this study sought to assess the seroprevalence for SARS-CoV-2 antibodies among a population of children who were admitted to hospital in Saudi Arabia. It also sought to determine whether the seroprevalence was associated with age group or gender.

## 2. Materials and Methods

### 2.1. Study Design, Setting, and Population

This is a cross-sectional study, the results of which are reported in accordance with the Guidelines for Strengthening the Reporting of Observational Studies in Epidemiology (STROBE) for Cross-sectional Studies [17]. The study was approved by the institutional review board of King Khalid University (approval number ECM#2021-5819), Abha, Saudi Arabia. The target population for this study is children attending Abha Maternity and Children Hospital (AMCH), a tertiary referral hospital in southwestern Saudi Arabia. The children who needed a blood test for any reason were included in this study. Written informed consent was obtained from all parents/guardians of the enrolled children. Inpatient, outpatient, and emergency department patients were included, based on two inclusion criteria: age less than 12 years of age and the need for a blood test for any reason. Patients were excluded for having risk factors for ongoing SARS-CoV-2 infection (typical symptoms, recent positive nasopharyngeal swab, contact with confirmed or suspected cases, and traveling abroad) and/or immunodeficiency, either primary or secondary. A total number of 413 children, who met the inclusion criteria, were sampled from the Serology Department at AMCH. Study data were obtained for patients seen between 1 October and 30 November 2021. An asymptomatic SARS-CoV-2 infection was defined as evident seropositivity in the absence of reported symptoms suggestive of COVID-19 (fever, chills, myalgia, ageusia, fatigue, anosmia, cough, and shortness of breath) [18].

### 2.2. Sampling Procedure and Processing

A sample of 454 children was planned to be included in the present study. The sample size was estimated based on an expected seroprevalence of 18% of COVID-19 antibodies in children [19], with an acceptable margin of error of 5% at a 95% confidence level and a design effect of 2. The sample size was determined using the Epi-Info 7 software (Centers for Disease Control and Prevention (CDC) in Atlanta, Georgia (US)), Serum samples were screened for SARS-CoV-2-specific IgG, IgM, and IgA antibodies using a commercially available indirect enzyme-linked immunosorbent assay (ELISA; Diapro, Milano, Italy). Two hundred microliters of the diluted (1:20 for IgM and 1:40 for IgG and IgA) test serum was added to microtiter plates containing 50 µL of neutralizing solution for IgM, 50 µL DILAS solution (the used assay diluent) for IgG and IgA along with a negative control in triplicate. A single positive control was added to the corresponding wells in the plates. One well was used as a blank in all plates for substrate addition only. The plates were sealed and incubated at 37 °C for 45 min. Plates were removed from the incubator and washed five times using an automatic ELISA plate washer 50 TS (BioTek^®^ Instruments, Inc. Winooski, Vermont, USA). Then, 100 µL of anti-IgG, IgM, and IgA horseradish peroxidase conjugates was added to the corresponding plates. The plates were sealed and incubated again at 37 °C for 45 min. The plates were then rewashed as 100 µL of the substrate solution was added to all the wells and left for 15 min at room temperature, followed by 100 µL of stop solution. Wells were read at 450/620 nm using the ELISA reader (Humareader, Human company, Wiesbaden, Germany). The results were calculated as an antibody ratio in relation to standard cut-off values.

### 2.3. Statistical Analysis

The data were collected, managed, coded, and entered into the statistical software IBM SPSS version 22 (SPSS, Inc. Chicago, IL, USA). Frequency and percent distributions were calculated for all variables, including demographic data, clinical characteristics, and comorbidities. In addition, the mean with standard deviation was calculated for normally distributed quantitative variables, while the range and median with interquartile range (IQR) were calculated for skewed numerical variables. Adjusted seroprevalence among the studied children was calculated in addition to crude prevalence to account for screening test sensitivity and specificity as the test validity measures are not 100% with some probability for false-positive and false-negative results [20]. An analysis was conducted to test whether there was a relationship between serological findings and both age and gender. Finally, a logistic regression was used to test the odds of being seropositive according to age and gender.

## 3. Results

### 3.1. Characteristics of the Study Sample

Between 1 October and 30 November 2021, SARS-CoV-2-specific IgG, IgM, and IgA antibody levels were measured in 413 samples. The demographics and clinical characteristics of the enrolled children are shown in Table 1. Of the 413 children who were enrolled, 243 (58.8%) were male. The age range of enrolled patients was between newborn and 12 years of age, with a median age of three years (interquartile range, 1–7) years. School-age children (older than 5) were the most common age category (40%), and all age intervals were represented. Most of the children had Saudi citizenship (390, 94.4%). The inpatient department was the most common source for the analyzed samples. More than two thirds of the enrolled patients had comorbidities (342; 82.8%), with cardiopulmonary symptoms being the most frequently reported comorbidities.

### 3.2. SARS-CoV-2 IgG, IgM, and IgA Seroprevalence Results

**Figure 1:**
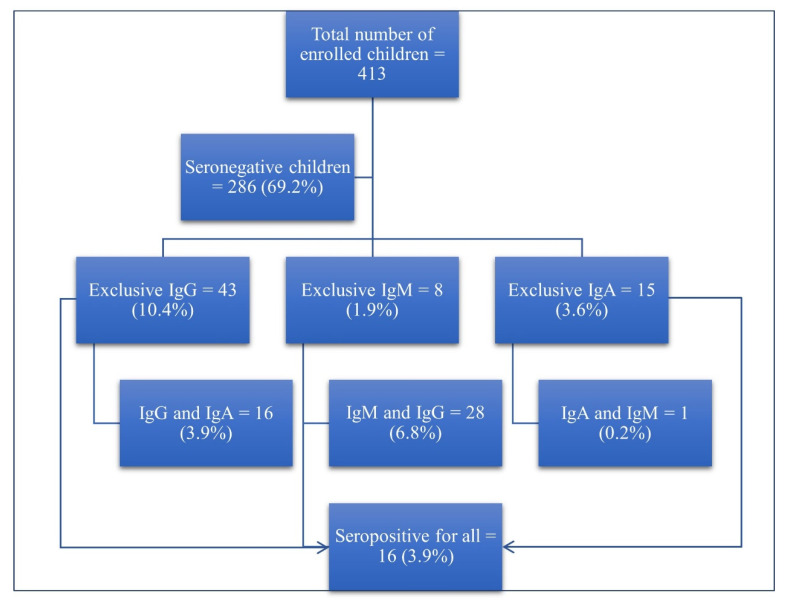
Flowchart of the seroprevalence among the study population.

We identified 127 (30.7%) seropositive children out of the total of 413 children tested. As for seropositive children, IgG was exclusively positive in 43 (10.4%) children; IgM was exclusively positive in 8 (1.9%) children, and IgA was exclusively positive in 15 (3.6%) children. Sixteen (3.9%) were IgG- and IgA-positive; 28 (6.8%) were IgM- and IgG-positive; 1 (0.2%) was IgA- and IgM-positive, and 16 (3.9%) were IgM-, IgG- and IgA-positive (Figure 1).

Table 2 shows the crude seroprevalence and adjusted seroprevalence of COVID-19 antibodies among the children from whom the samples were tested. The adjusted prevalence for IgG antibodies was 26.6% (95% CI: 22.3–30.9%); that for IgM antibodies was 13.6% (95% CI:10.2–16.8%), and IgA antibody prevalence was 12.4% (95% CI: 9.2–15.6%). In total, seropositivity was found among 33.0% (95% CI: 28.5–37.4%) of the children from whom the samples were obtained.

### 3.3. The Relation between Children’s COVID-19 Seropositivity and Their Age and Gender

Table 3 shows the crude and adjusted seroprevalence values of COVID-19 of children by their age and gender. The highest adjusted seroprevalence was found for children aged between 1 and 5 years 36.0% (95% CI: 28.4–43.6%), followed by school-age children (older than 5) 33.9% (95% CI: 26.7–41.1%) and children less than one year old 26.2% (95% CI: 17.3–35.1%). The adjusted seroprevalence was 35.9% (95% CI: 28.7–43.1%) among males and 30.8% (95% CI: 25.0–36.6%) among females.

Table 4 shows the crude and adjusted odds of the seropositivity of COVID-19 among children comparing males vs. females and different age groups. The odds of seropositivity adjusted for gender, age, and comorbidities was 1.54 (95% CI 0.86–2.74) for the 1–5-year-old age group compared to the children younger than 1 year old. Additionally, the rate of seropositivity for the age group >5 years was 1.41 (95% CI 0.79–2.5) compared with the children less than 1 year, while the rate of seropositivity among males was 1.25 (95% CI 0.8–1.87) compared to females. None of these comparisons was statistically significant. The crude odds ratios did not differ substantially from the adjusted odds ratios.

## 4. Discussion

This study evaluated the seroprevalence of SARS-CoV-2 in 413 nonvaccinated children. To the best of the authors’ knowledge, this is the first study on SARS-CoV-2 seroprevalence among children in southwest Saudi Arabia. Studies conducted among adult populations reported seroprevalence rates ranging from 1.4% to 50.2%. Other adult studies have also found different seroprevalence values [10,21,22,23,24]. This study was conducted before the Saudi MoH offered the COVID-19 vaccine for children between 5 and11 years of age, starting in February 2022. In southwest Saudi Arabia, we demonstrated a 33.0% adjusted seroprevalence rate of SARS-CoV-2 infection among hospital-based, nonvaccinated children aged 0–15 years. This result indicates that at least one in three unvaccinated children had acquired either symptomatic or asymptomatic COVID-19 infection within 18 months of the pandemic due to the presence of positive antibodies. The overall seroprevalence rate of SARS-CoV-2 among children in this study was higher than in other studies [13,14,15,16,19]. These previous studies showed a prevalence of 1.3% in Northern Cyprus [12], 5.8% among school- and preschool-age children in Canada [13], 9.5% to 15.4 (for IgG) in Italy [14,15], 27.1% in Saudi Arabia [19], and 25.3% in Brazil [16]. The higher prevalence in the current study could be explained by the fact that we conducted the study 18 months after the first reported case of COVID-19 in Saudi Arabia. The increase in seroprevalence in our study was consistent with the trend in confirmed cases in Saudi Arabia, with pediatric cases younger than 12 years of age increasing from 3% to 11% over a one-month period in May 2020 [5].

The SARS-CoV-2-specific IgG rate was 26.6%. In contrast, SARS-CoV-2-specific IgM and IgA were observed to be lower in the included children (13.6% and 12.4%, respectively). This indicates that a large number of children had previously been exposed to SARS-CoV-2. On the other hand, the presence of IgM and IgA suggests that many of the children may have had asymptomatic SARS-CoV, which is the most common manifestation of COVID-19 in children [24,25]. This is especially true as confirmed in previous studies, for the IgM and IgA classes of immunoglobulins specific to SARS-CoV-2 peak after two weeks following exposure to the virus, while the IgG peaks approximately at 28 days of infection [24,25,26,27].

The children who tested positive for SARS-CoV-2 IgM are suspected to have been infected by the virus in the period immediately prior to study since no vaccine is routinely used in this age group. No clinical disease was detected in these children on clinical observation, and consequently asymptomatic infection is suggested. An asymptomatic infection with SARS-CoV-2 is well-known and documented in various reports with an estimated prevalence of 30–50% of infections resulting in asymptomatic infection [24,25,28,29].

When comparing our study results with broad international studies that evaluated the seroprevalence of SARS-CoV-2 in children, the asymptomatic seropositivity rate found in our study is higher than the previously reported global seroprevalence, particularly within the first year of the pandemic, which was between 1 and 15% [5,12,13,14,15,16,20,21,30,31,32]. This variation may be explained by the fact that seropositivity is markedly influenced by various factors. These factors include variations in the study times and settings, sociodemographic criteria of the study participants, exposure risk, and implementation of school closures.

Although differences by age were not statistically significant, the current study revealed that children aged 1 to 5 years had the highest seroprevalence of SARS-CoV-2 (36.0% (28.4–43.6%)) compared to the other age groups of children tested. These children are considered to be in the preschool age group. This raises the issue that more attention should be paid to preventive care in this age group. In contrast, tested children younger than 1 year old had a seroprevalence of 26.2% (17.3–35.1%). Additionally, the study found a higher seroprevalence among males compared to females; however, this difference was not statistically significant. This finding is consistent with at least one other seroprevalence study from Saudi Arabia, which found a similar elevated seroprevalence among males compared to females [19]. While in this earlier study of adults, the higher seroprevalence may have been due to more exposure, such as work-related exposure among the male population, the reason for the increased seroprevalence among males in a pediatric population deserves more attention. A study from the United States estimated similar seroprevalence values for the population under 18 years of age for males and females [33].

Overall, the findings of the current study have public health implications, highlighting the importance of developing evidence-based guidelines for school closures and other social activities related to childhood during pandemics. This high seropositivity in children may provide a more accurate estimate of asymptomatic infections, which is needed to assess the spread of SARS-CoV-2 infection and gain a better understanding of local outbreaks of SARS-CoV-2 and how to control those outbreaks in the future. Pediatric hospitals may also need to implement more strict policies regarding infection control and the reduction in nosocomial infections since between 10 and 16% (IgM-positive) of visiting or admitted children had active asymptomatic infections with SARS-CoV-2.

Our study is limited by its hospital-based cross-sectional design. This design included patients with multiple comorbidities who needed frequent hospitalizations and were possibly exposed frequently to nosocomial infection. This may have led us to slightly overestimate the actual seroprevalence in the general child population. Data collection may also have suffered from a possible bias in the information collected from the medical records. Given the aforementioned limitations, we recommend community-based studies for the real estimation of the actual asymptomatic infection rate among the pediatric population in the country. In addition, the test used in this study does not have perfect sensitivity and specificity; although we adjusted for this issue in our analysis, it is still possible that some of those who tested positive for the presence of antibodies are false positives, and some of those who test negative for the presence of antibodies are false negatives. Furthermore, the presence of antibodies may decline over time; some children who were infected with SARS-CoV-2 early in the pandemic may not have tested positive because they no longer had detectable levels of antibodies. Moreover, SARS-CoV-2 antibodies have cross-reactivity with other viruses, such as MERS-CoV [8]. Some of the positive cases in this study may be due to infection with a virus other than SARS-CoV-2. Finally, the sample size of this study (413) was slightly below the required number based on our sample size calculation (454). This lower sample size may have impacted the precision of some of the estimates in this study and our ability to determine whether differences were statistically significant.

## 5. Conclusions

This is the first study identifying the seroprevalence of SARS-CoV-2 among a hospital-based pediatric population in the southwest region of Saudi Arabia. The observed rates likely reflect the patient population served by Abha Maternity and Children Hospital and do not necessarily apply to other centers. However, this study sheds light on the dynamics of viral transmission in the community of southwestern Saudi Arabia. It provides a good reference for future studies because of the large number of cases investigated, especially as future studies will be influenced by antibodies formed in response to vaccination. Future research should examine factors related to SARS-CoV-2 infection in pediatric populations and any protection afforded by the presence of SARS-CoV-2 antibodies from a previous infection.

## Figures and Tables

**Table 1:** Demographics and clinical characteristics of the enrolled children.

Clinical Characteristic	Study Group n (%)
Age, median [IQR; min–max]	3 [1–7; 0–12]
Age categories in years	
<1	94 (22.8)
1–5	152 (36.8)
>5	167 (40.4)
Gender	
Female	170 (41.2)
Male	243 (58.8)
Citizenship	
Saudi	390 (94.4)
Foreign	23 (5.6)
Department	
Outpatient	81 (39.5)
Emergency room/PICU	10 (4.9)
Inpatient	114 (55.6)
Comorbidities	
Yes	342 (82.8)
No	71 (17.2)

Data are shown as the median (interquartile range) or number (%) as appropriate. Pediatric Intensive Care Unit, PICU.

**Table 2:** Crude and adjusted seroprevalence values of COVID-19 antibodies among children in Aseer region, Saudi Arabia.

Seroprevalence	No.	Crude Prevalence	Adjusted Prevalence	95% CI
**IgG**	103	24.9%	26.6%	22.3–30.9%
**IgM**	53	12.8%	13.6%	10.2–16.8%
**IgA**	48	11.6%	12.4%	9.2–15.6%
**Seropositive**	127	30.8%	33.0%	28.5–37.4%

CI: confidence interval.

**Table 3:** Crude and adjusted seroprevalences values of COVID-19 antibodies among children by their age and gender the in Aseer region, Saudi Arabia.

Variable	No.	Crude Prevalence (95% CI)	Adjusted Prevalence (95% CI)
**Age in years**			
**-** **<1**	23	24.5% (15.2–33.8%)	26.2% (17.3–35.1%)
**-** **1–5**	51	33.6% (25.4–40.3%)	36.0% (28.4–43.6%)
**-** **>5**	53	31.7% (24.6–38.7%)	33.9% (26.7–41.1%)
**Gender**			
**-** **Male**	70	55.1% (25.8–42.1%)	35.9% (28.7–43.1%)
**-** **Female**	57	44.9% (23.4–34.6%)	30.8% (25.0–36.6%)

CI: confidence interval.

**Table 4:** Adjusted and crude relation between children’s COVID-19 seropositivity and their age and gender.

Factors	COR	95% C.I for OR		AOR	95% C.I for OR	
		Lower	Upper		Lower	Upper
**Age 1–5 Yrs. vs. <1 Yr.**	1.56	0.874	2.779	1.54	0.86	2.74
**Age > 5 Yrs. vs. <1 Yr.**	1.44	0.810	2.543	1.41	0.79	2.5
**Male vs. Female**	1.25	0.82	1.89	1.20	0.80	1.87

COR: crude odds ratio; AOR: adjusted odds ratio, adjusting for age, gender, and the presence of commodities; CI: confidence interval.

## Data Availability

The datasets used in this study are available from the corresponding authors upon request.
